# The 27 kDa *Trypanosoma brucei* Pentatricopeptide Repeat Protein is a G-tract Specific RNA Binding Protein

**DOI:** 10.1038/s41598-018-34377-9

**Published:** 2018-11-19

**Authors:** Pakoyo F. Kamba, David A. Dickson, Neil A. White, Jennifer L. Ekstrom, Donna J. Koslowsky, Charles G. Hoogstraten

**Affiliations:** 1 Department of Biochemistry and Molecular Biology, Michigan State University, East Lansing, Michigan 48824-1319 USA; 2 Graduate Program in Cell and Molecular Biology, Michigan State University, East Lansing, Michigan 48824-1319 USA; 3 Department of Microbiology and Molecular Genetics, Michigan State University, East Lansing, Michigan 48824-1319 USA; 4 Makerere University, Kampala, Uganda; 5 Present Address: Sackler School of Graduate Biomedical Sciences, Tufts University, Medford, Massachusetts USA; 6 Present Address: Department of Molecular, Cellular, and Developmental Biology, Yale University, New Haven, Connecticut USA; 7 Present Address: Department of Food Science and Human Nutrition, Michigan State University, East Lansing, Michigan USA

## Abstract

Pentatricopeptide repeat (PPR) proteins, a helical repeat family of organellar RNA binding proteins, play essential roles in post-transcriptional RNA processing. In *Trypanosoma brucei*, an expanded family of PPR proteins localize to the parasite’s single mitochondrion, where they are believed to perform important roles in both RNA processing and translation. We studied the RNA binding specificity of the simplest *T*. *brucei* PPR protein (KRIPP11) using electrophoretic mobility shift assays, fluorescence anisotropy, circular dichroism spectroscopy, and *in vitro* selection. We found KRIPP11 to be an RNA binding protein with specificity for sequences of four or more consecutive guanosine residues (G-tracts). Such G-tracts are dramatically enriched in *T*. *brucei* mitochondrial transcripts that are destined for extensive uridine insertion/deletion editing but are not present in mRNAs following editing. We further found that the quadruplex oligoguanosine RNA conformation is preferentially recognized by KRIPP11 over other conformational forms, and is bound without disruption of the quadruplex structure. In combination with prior data demonstrating association of KRIPP11 with the small ribosomal subunit, these results suggest possible roles for KRIPP11 in bridging mRNA maturation and translation or in facilitating translation of unusual dual-coded open reading frames.

## Introduction

Trypanosomes, including the causative agent of human African trypanosomiasis (HAT) *Trypanosoma brucei*, are characterized by a single, large mitochondrion (kinetoplast) containing a disc-shaped DNA genome, itself made from a concatenated network of large DNA maxicircles and small DNA minicircles^1,2^. This unusual mitochondrion is central to the life cycle and pathogenesis of kinetoplastid parasites. In *T*. *brucei*, modulation of mitochondrial activity facilitates parasite adaptation from the sugar-deficient tsetse fly gut, where it depends on oxidative phosphorylation, to the sugar-rich environment in human blood, where it depends on glycolysis for energy^3,4^. In these parasites, both nuclear and mitochondrial genes are polycistronic and most regulation of gene expression occurs at the RNA level^5–9^. Strikingly, 12 of the 18 protein coding genes in trypanosome mitochondrial DNA are composed of incomplete open reading frames (ORFs) which are post-transcriptionally converted into translatable mRNAs by a process of systematic uridine insertion and/or deletion called RNA editing^10^. In fact, the primary (pre-edited) transcripts of nine of the *T*. *brucei* mitochondrial mRNA genes undergo such massive editing (pan-editing) that approximately 50% of the sequence of mature mRNA consists of externally inserted uridines^10,11^. RNA editing by nucleotide insertion and deletion is unique to trypanosomes^12^, and thus serves as a potential target for pharmacological intervention.

RNA editing and other post-transcriptional RNA processing events in trypanosome mitochondria are executed by a variety of RNA binding proteins whose roles and mechanisms of action are only now being characterized^10,13–17^. Deciphering the RNA binding characteristics of these important proteins is therefore critical to our understanding of the relationship between specific RNA targeting and biological function. Among trypanosome mitochondrial RNA binding proteins, the pentatricopeptide repeat (PPR) family of sequence-specific RNA binding proteins has proven to be particularly important. First discovered in *Arabidopsis thaliana* in 2000, PPR proteins are characterized by tandem repeats of 35 amino acids, with each motif folding into a pair of antiparallel alpha-helices similar to the peptide-binding tetratricopeptide (34 amino acid) repeat (TPR) motif^18,19^. Over the last decade, these proteins have been linked to a wide variety of stages of post-transcriptional RNA processing in chloroplasts and plant mitochondria^20–24^. Typically, plant genomes code for hundreds of PPR proteins whereas non-plant eukaryotes contain fewer than ten^25^. An exception is found in trypanosomes, where approximately 40 PPR proteins have been reported in *T*. *brucei*^14,26,27^. These proteins vary dramatically both in the number of PPR repeats and in the presence and nature of accessory non-PPR domains. PPR proteins are highly conserved across trypanosomatids and RNAi knockdowns cause parasite growth retardation, growth arrest, and death, consistent with functional essentiality^16,26–28^. Underlying the severe phenotypes is deterioration in oxidative phosphorylation and thus in mitochondrial function^16,26–29^.

Proteomic and genetic analyses have provided some insights into the functions of *T*. *brucei* PPR proteins. Most *T*. *brucei* PPR proteins co-purify with polyadenylation complexes and/or mitochondrial ribosomal subunits, suggesting a role in their respective functions^29–32^. For PPR proteins in which the effect of RNAi on mRNA processing has been reported, namely, KPAF1, KRIPP1 and KRIPP8, compromised poly(A/U) synthesis and translation of mitochondrial mRNA has been observed^29,32^. However, knockdown of different PPR proteins often affects specific post-transcriptional processes and specific gene transcripts^26–29,32^, suggesting their cognate RNA sequences are unique.

Identifying the specific RNA ligands for PPR proteins is crucial to elucidating their mechanism(s) of RNA binding. Unfortunately, poor protein solubility and difficulty in heterologous expression^25,33^ have limited such studies to a restricted subset of plant PPR proteins. These studies have shown that PPR proteins exhibit high sequence specificity in RNA recognition. For example, maize PPR10 protects and defines both the 5′ and 3′ termini of transcripts from two different loci (*atpH* and *psaJ*) by binding to a highly conserved RNA motif located in the intergenic regions downstream of each gene^34,35^. Another PPR protein, CRR4 from *Arabidopsis thaliana*, specifies the site of RNA editing in the chloroplast *ndhD* gene by recognizing a specific motif surrounding the editing site^24,36^. In maize, PPR103 protects the 5′-terminus of processed *rpl16* transcripts from exonucleolytic degradation^37^. Numerous examples of this type have led to a general picture of plant PPR proteins as sequence-specific effectors and modulators of specialized organellar RNA metabolism^23,38^.

For the plant PPR proteins whose specific RNA ligands are known, progress has been made in elucidating their RNA binding mechanisms via biochemical studies with truncated PPR proteins, computational modeling, and x-ray crystallography. Biochemical studies have shown retention of RNA binding in severely truncated proteins, suggesting that the RNA binding activity resides in the PPR modules. Computational studies by phylogenetic analysis of residue conservation across numerous PPR motifs^39^ and iterative alignment of residues at each position in the PPR motif with bases in the cognate RNA footprint have suggested a common code of RNA binding in which each PPR repeat recognizes a single nucleotide via RNA contacts from amino acids at positions 1, 3 and 6^40–42^. Experimental studies using engineered PPR proteins have provided strong support for this mechanism of single-stranded RNA recognition^43^. Crystal structures of two plant-derived PPR proteins in complex with RNA targets (maize PPR10 and *Arabidopsis thaliana* THA8) confirmed the recognition of individual nucleotides of single-stranded RNA by individual PPR repeats and elucidated the basis for many aspects of the emerging recognition code^44–46^. These correspondences have been more finely mapped by a set of four crystal structures of designed PPR proteins (dPPRs) in complex with their predicted RNA targets^47^.

**Figure 1:**
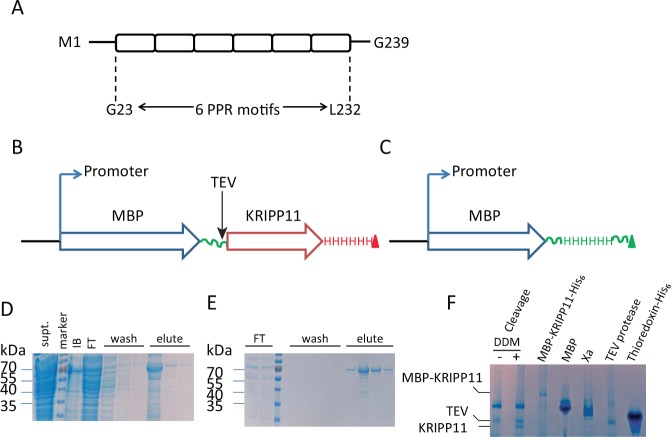
(**A**) Organization of PPR motifs in KRIPP11. (**B**) Scheme of the overexpression construct for the MBP-KRIPP11 fusion protein emphasizing the His_6_ tag at the C-terminus of KRIPP11. TEV protease cleavage site, which leaves two additional residues (GS) at the N-terminus of KRIPP11, is indicated. (**C**) Overexpression construct for free MBP with a His_6_ tag at the C-terminus. (**D**) SDS-PAGE for MBP-KRIPP11-His_6_ fractions from the Ni-NTA column. Supt., supernatant; m, PageRuler™ size marker; IB, inclusion bodies; FT, flow through; three lanes of wash and three of eluate. (**E**) SDS-PAGE of MBP-KRIPP11-His_6_ fractions from the amylose column. FT, two lanes flow through; m, marker; four lanes of wash and four of eluate. (**F**) 6–12% gradient native polyacrylamide gel showing the monomeric state of KRIPP11. TEV cleavage mixture is shown in the absence and presence of DDM detergent (see text). For this and subsequent figures, uncropped originals of all gel images are available as Supplementary Information (Fig. S2).

In contrast to plants, the specific RNA sequences recognized by individual *T*. *brucei* PPR proteins have hardly been elucidated, hindering progress in their mechanistic studies and subsequent biotechnological applications. Trypanosomal PPR proteins vary greatly in the number of PPR repeats and in the number and nature of accessory domains. In the initial work reported here, we focused on the 27 kDa *T*. *brucei* PPR protein KRIPP11 (TriTrypDB Tb927.8.6040; genbank XM_842341; previously PPR27), which is one of the smallest and simplest members of the protein family. The four PPR repeats originally identified in this protein^26^, together with two additional 35-residue regions predicted to carry strong helical content, yield a probable total of six tandem PPR repeats in 239 amino acids and no identifiable accessory domains (Fig. 1A). Though KRIPP11 was initially thought to be a cytoplasmic protein due to negative mitochondrial targeting by classical computational tools^27^, contemporary computational tools support mitochondrial localization (Supplementary Material, Table S1). Mitochondrial localization is experimentally supported by a strong kinetoplast signal in imaging data from the TrypTag database^48^ and by the demonstration of stable RNA-independent association with mitochondrial small subunit ribosomal complexes^31,32^. In this work, we use an integrated approach of biophysical probing of RNA affinity using electrophoretic shift mobility assay (EMSA) and equilibrium fluorescence anisotropy and *in vitro* selection of protein ligands to derive significant insight into the binding specificity and function of this protein. We find that KRIPP11 shows a strong preference for sequences containing G-tracts of four to six residues or more, such as are extensively found in the subset of kinetoplastid pre-mRNAs destined to undergo extensive uridine insertion/deletion editing. Preliminary *in vitro* evidence suggests targeting of G-tracts in quadruplex RNA form.

## Results

### Heterologous expression of soluble, monomeric KRIPP11

KRIPP11 was expressed as a fusion protein with maltose binding protein (MBP) to promote yield and solubility. Using the constructs in Fig. 1B,C, we obtained a mean soluble yield of 5.0 mg per liter of culture for the MBP-KRIPP11 fusion protein. After tandem affinity chromatography using sequential Ni-NTA and amylose resins, fusion protein of over 90% purity was obtained (Fig. 1D,E). Following removal of the MBP tag with TEV protease, the oligomeric state of mature KRIPP11 was verified using native PAGE. The mature KRIPP11 migrated slightly faster than the 27 kDa TEV protease, consistent with a monomeric state of this 24.4 kDa protein in the cleavage mixture (Fig. 1F). Since the cleaved KRIPP11 was found to be vulnerable to aggregation and precipitation upon purification, uncleaved MBP-KRIPP11-His_6_ fusion protein was used for the binding assays and selections. Separately expressed MBP-His_6_ (Fig. 1C) was used as a control to rule out MBP as the source of observed nucleic-acid binding.

### Binding specificity of KRIPP11 for single-stranded poly(G) RNA

**Figure 2:**
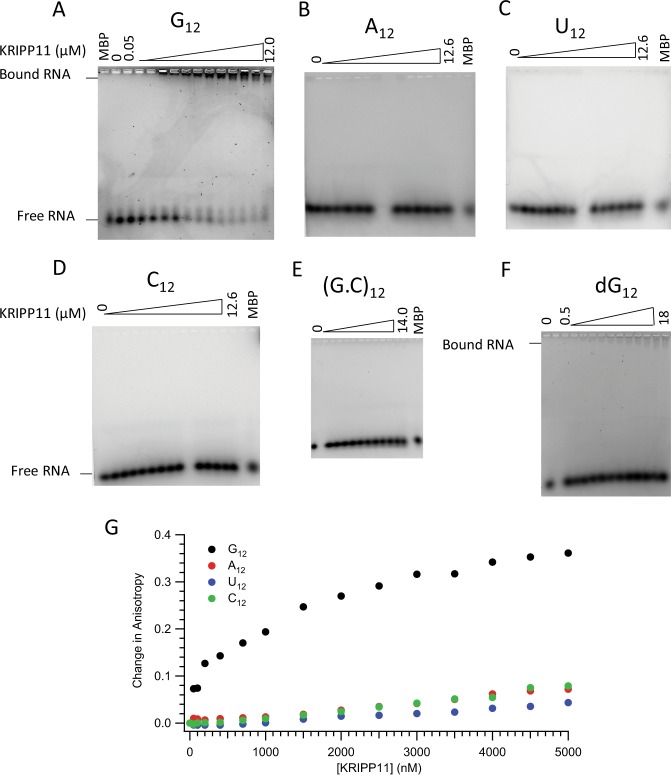
Analysis of KRIPP11 binding. (Panels A–D) EMSA analysis on 2% tris-glycine agarose gels showing retardation of G_12_ and not the other RNA homooligomers (A_12_, U_12_, and C_12_) as a function of protein concentration; (**E**) (G.C)_12_; (**F**) dG_12_. A reaction with MBP-His_6_ was used as a negative control for EMSA experiments. (**G**) Fluorescence anisotropy binding isotherms for KRIPP11 against ssRNA homooligomers.

Preliminary insights into the sequence- and chemical specificity of KRIPP11’s nucleic acid ligand were obtained by assessing the protein’s affinity for homooligomeric and simple heterooligomeric RNAs and DNA. Among 12-nucleotide homooligomers, EMSA showed an upward shift in the mobility of 12-nucleotide ssRNA for only poly(G) for protein concentrations up to 12 μM (Fig. 2A–D).

**Figure 3:**
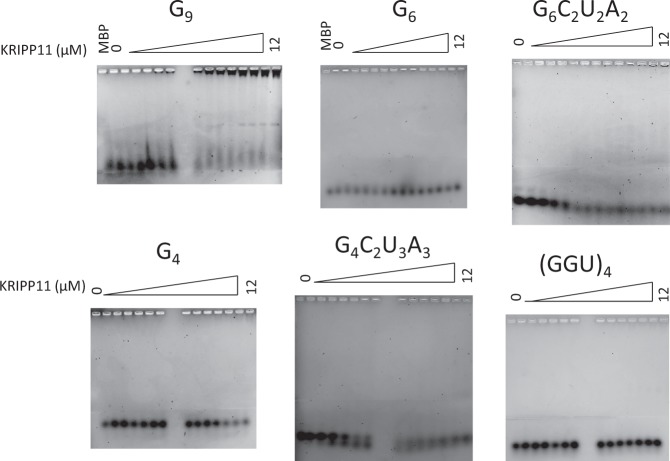
EMSA of KRIPP11 with G-tracts varying in length and sequence.

We further explored the binding specificity of KRIPP11 for single- versus double-stranded RNA as well as versus the identical DNA sequence. Annealing of G_12_- with an excess of C_12_-RNA to form double-stranded (G.C)_12_ abolishes the KRIPP11-induced upward shift in poly(G) mobility (Fig. 2E). By contrast, an interaction with G_12_ ssDNA is observed, although only at higher concentrations of protein, indicating a reduced binding affinity (Fig. 2F). Finally, we tested the affinity of KRIPP11 for shorter G homooligomers as well as representative G-rich heterooligomers and a (GGU)_4_ sequence suggested by the *in vitro* selection results (see below). Of the sequences tested, intense shifted bands on increasing KRIPP11 concentration only appeared with the G_9_ homooligomer (Fig. 3). EMSA experiments testing G_12_, G_9_, and G_6_ against MBP-His_6_ showed no evidence of binding, confirming that the observed high-affinity binding is to the KRIPP11 moiety rather than to the MBP or His_6_ fusion partners (“MBP” lanes in Figs 2 and 3). The EMSA results thus emphasize that, among the variety of sequences examined, stable KRIPP11-RNA complexes are only observed for longer poly(G) sequences.

**Figure 4:**
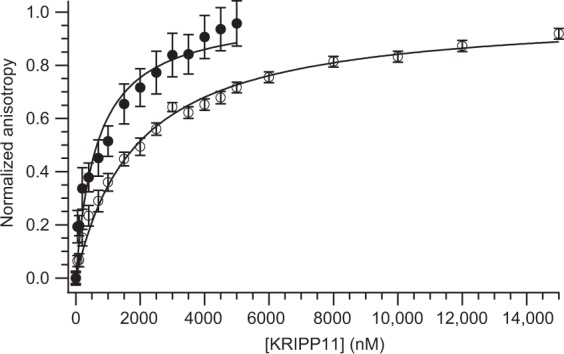
Determination of the K_d_ for interaction of KRIPP11 with G_12_ ssRNA (solid circles) and G_12_ ssDNA (open circles) from fluorescence anisotropy data. Curves represent fits to a single-site binding isotherm (Equation 2).

For quantitative determination of equilibrium binding constants *K*_d_, we applied a fluorescence anisotropy binding assay. In this technique, the reorientation of a fluorophore bound to the nucleic acid oligomer is slowed upon protein complex formation due to slower global tumbling, leading to a greater retention of polarization in fluorescence emission upon excitation with a polarized source. Examination of titrations for the 12-mer homooligomers showed a substantial, saturatable shift in anisotropy only for G_12_ (Fig. 2G), consistent with the EMSA results (Fig. 2A–D). Upon fitting to a single-site isotherm (Equation 2, Methods), a *K*_*d*_ of (0.66 ± 0.13) µM was obtained for G_12_, increasing to (1.88 ± 0.13) μM for the corresponding deoxy sequence (Fig. 4). The binding curve saturated and fit well to the standard isotherm, consistent with a specific interaction. Two independent measures of binding thus confirm the specificity of KRIPP11 for poly(G) among the homooligomers.

By contrast, poly(G) sequences as short as G_4_ and a subset of the heteropolymers tested displayed saturable, specific interactions via fluorescence anisotropy assays, albeit in all cases with reduced affinity compared to G_12_, despite the lack of complex formation in EMSA (Supplementary Material, Fig. S1). Dissociation constants for measured sequences are collected in Table 1. Affinity tracked roughly with the longest stretch of consecutive G residues; for example, (GGU)_4_ displayed the weakest affinity of the sequences analyzed despite having a relatively high number (eight) of total G residues. The contrast between anisotropy data and EMSA results may be explained by the nature of EMSA as a non-equilibrium assay, which makes it poorly suited to the detection of interactions with a fast ligand dissociation rate. On the other hand, FA is an equilibrium assay with greater dynamic range of analysis for binding affinity than EMSA^49^. The quantitative results in Table 1 reiterate the nature of KRIPP11 as a nucleic acid binding protein with specificity for nonduplex G-tract RNA sequences.

**Table 1:** Affinity of KRIPP11 for RNA G-tracts of varying sequence determined using fluorescence anisotropy.

RNA	K_d_ (µM)
G_12_	0.66 ± 0.13
G_9_	1.87 ± 0.03
G_6_	1.07 ± 0.01
G_6_C_2_U_2_A_2_	0.93 ± 0.01
G_4_	3.42 ± 0.19
G_4_C_2_U_3_A_3_	2.21 ± 0.03
(GGU)_4_	6.55 ± 0.10

### *In vitro* selection of KRIPP11 binding sequences

**Figure 5:**
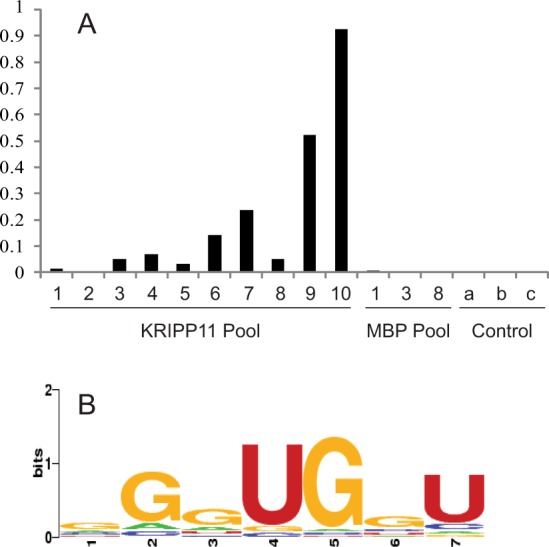
(**A**) RiboGreen assay showing increase in percentage of protein that bound RNA with successive cycles of *in vitro* selection with KRIPP11. After the second round of selection, the amount of RNA-bound protein was undetectable, attributed to an error in the assay. Control assays: a, protein-free beads in presence of RNA pool; b, protein-bound beads in absence of RNA pool; c, protein-free beads in absence of RNA pool. (**B**) WebLogo of RNA sequences sequenced following the final round of selection with KRIPP11.

To delineate the binding specificity of KRIPP11 in a more unbiased fashion, we applied the technology of *in vitro* selection to isolate RNA sequences with high affinity to the protein from a pool of over 10^18^ RNA sequences randomized at 20 continuous positions. *In vitro* selection of aptamer sequences from fully randomized pools such as this provides an unbiased view of consensus RNA binding preferences for proteins and other molecules^50,51^. During 11 rounds of *in vitro* selection, the RNA pool was progressively enriched with KRIPP11-binding activity as measured by the partitioning of fluorescent RNA into the protein-bound pool (Fig. 5A). Sequencing of 100 clones from the final selection round revealed a bound pool enriched in poly(G) (4 to 8 consecutive G nucleotides) and to a lesser extent poly(U) (4 to 5 consecutive U nucleotides) sequences (Table S2). Little specificity for specific sequences was obtained, as reflected in the relatively low-information consensus binding sequence produced by standard algorithms (Fig. 5B). Assay of binding to a repeating GGU sequence suggested by the apparent consensus sequence yielded weaker binding than a simple G homooligomer (Table 1). This lack of validation tends to argue against the functional significance of the U-containing motif, although effects arising from the somewhat different buffer conditions in use for the two procedures may also account for the discrepancy (see Methods; briefly, fluorescence polarization assays were conducted in 20 mM Tris-HCl pH 7.5, 0.15 M KCl, 5 mM MgCl_2_, 1 mM DTT, whereas the binding buffer for selection experiments was 50 mM phosphate pH 7.5, 0.1 M KCl, 1.5 mM MgCl_2_, 2 mM imidazole). Taken together with the homopolymer binding data above, the selection results establish KRIPP11 as a G-tract specific RNA binding protein.

### Potential functional role of a G-tract binding protein in *T*. *brucei*

**Figure 6:**
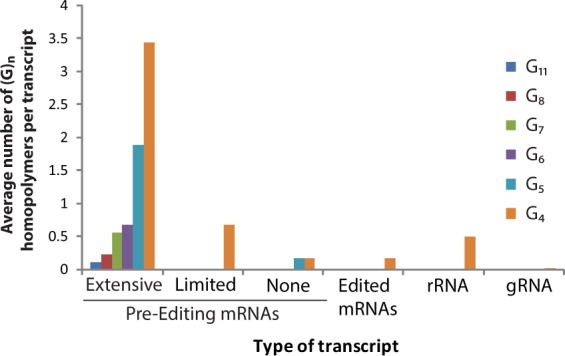
Prevalence of poly(G) homopolymers of 4, 5, 6, 7, 8, and 11 contiguous guanosine residues. The vertical axis reports the number of homopolymeric G sequences of exactly *n* residues found in the indicated family of transcripts divided by the number of transcripts in the family. Pre-editing mRNAs are divided into classes destined to undergo extensive editing, limited editing, or no editing (see text).

To investigate the potential biological significance of these results, we examined the *T*. *brucei* mitochondrial genome for open reading frames coding for G-rich pre-mRNAs. Interestingly, a strong pattern of preferential enrichment of G-tracts in some transcripts was observed, particularly with respect to the U-insertion/deletion editing state of the transcripts. Specifically, we divided pre-edited transcripts into nine that undergo extensive editing, with more than 50% of the final sequence consisting of inserted uridines (“pan-edited” henceforth); three that undergo editing events to a much less dramatic extent and exclusively to the 5′ end (Cyb and Murf II) or correction of an internal frameshift (COII) (“limited” editing); and the six mitochondrial transcripts that undergo no insertion/deletion editing (“never-edited”). The final versions of the twelve pan- and limited-editing transcripts after all editing events are complete are denoted “post-editing”. Of these four classes, as well as mitochondrial rRNA transcripts, the pan-edited transcripts were specifically and dramatically enriched in G-tracts of at least four consecutive guanosines. Indeed, all but one of the pan-edited transcripts contained at least one, and usually several, G-tracts of six or more consecutive guanosines, a pattern with zero occurrences in the other transcript classes (Fig. 6; Supplementary Material, Tables S3–S7). Further, all of the more than 60 G-tracts identified in pan-edited transcripts are destroyed by the U-insertion/deletion editing, and no new ones are created (cf. Tables S3 and S5); in an interesting contrast, the two four-nucleotide G-tracts present in transcripts destined for limited editing survive the editing process intact (cf. Tables S4 and S5). We also searched available libraries of insect- and bloodstream-form gRNA sequences from high-throughput sequencing^52,53^; an overall average of 0.22% of guide RNAs contained a G-tract of four or more nucleotides. Given that G-tracts of four to six or more consecutive nucleotides are necessary for stable KRIPP11 binding, the conclusion is that only pan-edited transcripts are likely to be bound by KRIPP11 *in vivo*, and only prior to the completion of the editing process.

### Preference of KRIPP11 for putative G-quadruplex forming sequences

**Figure 7:**
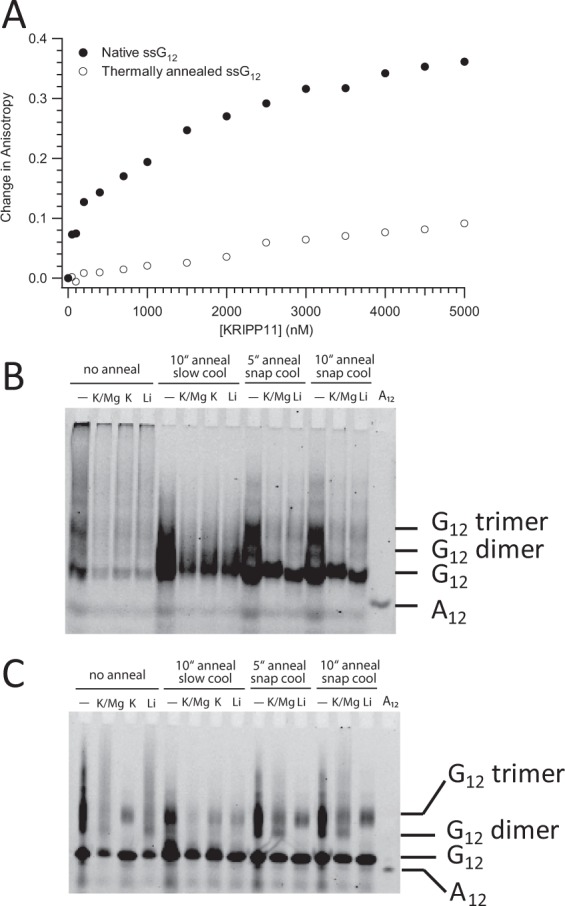
(**A**) Anisotropy titrations of native and thermally denatured G_12_ ssRNA with KRIPP11. (**B**) Native 18% polyacrylamide gel showing the electrophoretic profile of 5′-FLUO-G_12_ pre-exposed to different thermal histories and salt conditions. “No anneal” samples were purified under nondenaturing conditions and exchanged to the indicated buffer. For other samples, time of incubation in minutes at 95 °C and mode of cooling is indicated; see main text for details. K/Mg buffer, 20 mM Tris-HCl, pH 7.5, 150 mM KCl, 5.0 mM MgCl_2_, 1.0 mM DTT; K, 20 mM Tris-HCl, pH 7.5, 150 mM KCl, 1.0 mM DTT; Li, 20 mM Tris-HCl, pH 7.5, 150 mM LiCl, 1.0 mM DTT. A_12_ ssRNA was dissolved without annealing in buffer TE (10 mM Tris-HCl, pH 7.5, 0.1 mM EDTA). (**C**) Denaturing (6.8 M urea) 17% polyacrylamide gel with the same lane content as in B.

**Figure 8:**
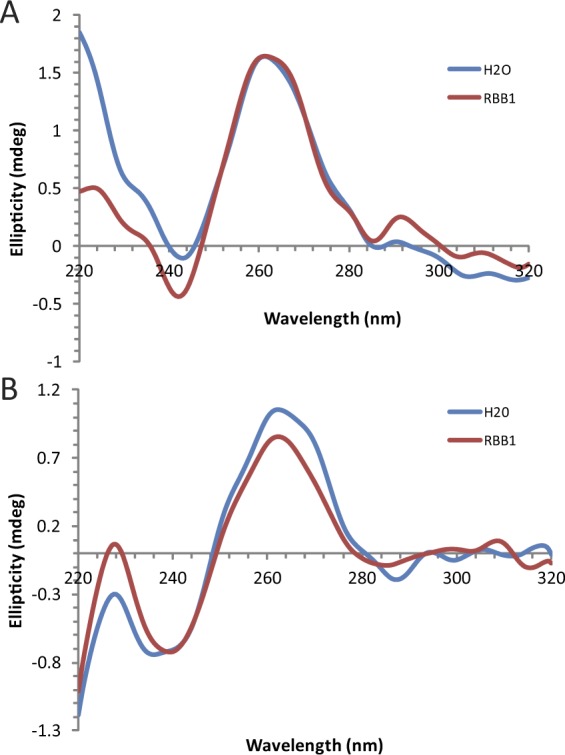
CD spectra of G_12_ (**A**) and G_4_ (**B**) in MQ-H_2_O (blue) and K/Mg buffer (brown). The minima at 245 nm and maxima at 265 nm are characteristic of parallel G-quadruplexes.

RNA sequences containing contiguous runs of guanosine residues can, under appropriate conditions, form intra- and intermolecular G-quadruplex structures^54–56^. Thus, two interesting questions for a G-tract specific binding protein such as KRIPP11 are whether it will selectively bind RNA in quadruplex form in solution and, if so, whether that structure is maintained in the protein-bound form. Since the formation of intermolecular G-quadruplexes by short G-rich oligos is likely to be dependent on the thermal history of the sample, we compared the binding of KRIPP11 to RNA homo-oligomers prepared under thermally denaturing and native conditions (see Methods). When RNA previously thermally denatured at 95 °C followed by snap cooling on ice was used in binding assays, the increase in anisotropy with KRIPP11 concentration was greatly diminished relative to native RNA and the binding isotherm did not saturate, consistent with weakened or less-specific binding (Fig. 7A). To assign the structural differences between denatured/renatured and natively-prepared RNA samples, we applied circular dichroism (CD) spectroscopy and native gel analysis. The CD spectra of native G_12_ and G_4_ ssRNA displayed minima at 245 nm and maxima at 265 nm (Fig. 8), consistent with a parallel G-quadruplex conformation^55,57^. On native PAGE, native G_12_ not only migrated as a ladder-like smear of various RNA sizes larger than that of A_12_ RNA, most of the RNA was also retained in the well (Fig. 7B), consistent with a multimeric state for these species. Following Tluckova and coworkers^58^, we speculate that that the slowest migrating native G_12_ RNA forms and those retained in the well are tetrameric quadruplexes and their aggregates, respectively. A denaturing gel of the G-tracts revealed the resistance of some quadruplexes to denaturation (Fig. 7C).

Interestingly, the intensity of native G_12_ bands on nondenaturing gels was severely diminished in the presence of monovalent ions compared to that of thermally treated RNA. Monovalent and divalent ions stabilize quadruplex structure^59,60^, and it is possible that they drive the G-tract RNA to a state in which the fluorescein stacks against the guanosine, thereby reducing its quantum yield. Thermally treated but slowly cooled G_12_ RNA migrates as a single elongated band at the speed of the fastest migrating band of native RNA (Fig. 7B). It is likely that the elongated band results from overlapping dimeric quadruplexes and monomeric RNA^58^, of which the former severely bleaches fluorescein in the presence of metal ions. In contrast, when thermally treated RNA is rapidly cooled in the absence of metal ions, up to 20% of G_12_ migrated at a size most likely to be a trimer while the rest migrated as monomers. In the presence of either K^+^ or Li^+^ ions under rapid cooling, however, this larger (trimer-like) band of G_12_ RNA diminished, likely due to transformation into more stable dimeric quadruplexes with concomitant quenching of fluorescein, similar to the non-thermally exposed RNA.

In short, KRIPP11 binds RNA with high affinity according to fluorescence polarization assays only if that RNA has a thermal history, specifically preparation under native conditions, that shows CD spectra and native-gel behavior consistent with a significant degree of G-quadruplex formation, suggesting that KRIPP11 binding is specific to quadruplex structures.

### KRIPP11 binding does not disrupt G-quadruplex structure

**Figure 9:**
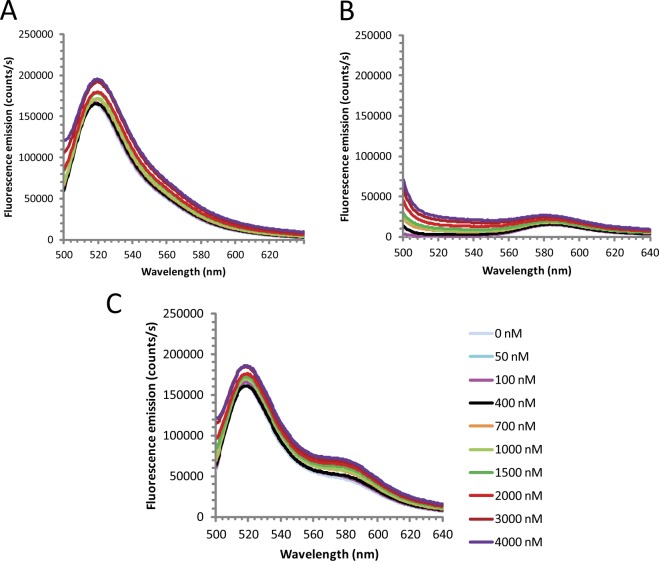
*Intermolecular FRET in G-tract RNA*. Fluorescence emission intensity of (**A**) 5′-FLUO-G_12_; (**B**) G_12_-TAMRA-3′; (**C**) An annealed mixture of FLUO-G_12_ and G_12_-TAMRA as a function of KRIPP11 concentration. Excitation was at 490 nm in all cases. Traces presented are the average of three replicate experiments.

Some proteins are known to remodel nucleic acid quadruplex structures upon binding^61–63^. To examine the state of KRIPP11-bound RNA, we applied fluorescence resonance energy transfer (FRET) analysis. We prepared a mixture of G_12_ RNA oligomers containing either a 5′-fluorescein (FLUO) or 3′-TAMRA fluorescent label. A strong FRET affect will be observed only if an intermolecular interaction such as G-quadruplex formation brings the fluorescein donor and TAMRA acceptor within the Förster radius (approximately 55 Å for this donor-acceptor pair), allowing efficient nonradiative donor-acceptor energy transfer. If KRIPP11 were to unfold the RNA quadruplexes, therefore, any FRET signal arising from the intermolecular quadruplex would reduce with KRIPP11 concentration. Here, we found that when annealed to 5′-FLUO-G_12_, the intensity of G_12_-TAM-3′ emission increases at least 3-fold relative to G_12_-TAMRA-3′ alone (Fig. 9A–C) following excitation at 490 nm. When the sum of the spectra of free 5′-FLUO-G_12_ and free G_12_-TAM-3′ was deducted from the spectrum of annealed 5′-FLUO-G_12_/G_12_-TAMRA-3′, a net positive TAMRA emission signal (peak intensity of about 8,000 counts/s) and no FLUO emission signal was observed, consistent with a FRET effect. Since the fluorescence donors and acceptors reside on different RNA chains, FRET arises unambiguously from the formation of intermolecular interactions, presumably in the form of G-quadruplex structures (cf. Figure 6). Specifically, the FRET signal arises solely from the subset of complexes containing both 5′-FLUO-G_12_ and G_12_-TAMRA-3′; complexes containing only multiple copies of a single species do not contribute to the observed transfer. The TAMRA emission intensity from FRET was only 5% of the emission intensity of free 5′-FLUO-G_12_, implying that there was low efficiency of resonance energy transfer. However, the low FRET signal was not unexpected as G-tracts without intervening heterogeneous nucleotides (A, U, and C) are very stable with melting points above 95 °C^60,64^. Thus, only a relatively small fraction of the RNA is unfolded and available to anneal to new partners.

The hypothesis that protein binding disrupts intermolecular quadruplex formation predicts that the magnitude of FRET would decrease upon protein-RNA complex formation. Upon titration with KRIPP11, however, the fluorescence emission of G_12_-TAM-3′ in the 5′-FLUO-G_12_/G_12_-TAMRA-3′ FRET combination (i.e., the FRET signal) not only was not reduced but indeed increased (Fig. 9C). Besides structural remodeling, a change in fluorescence emission intensity of a ligand-conjugated fluorophore upon protein binding can also occur if the interaction alters the microenvironment around the fluorophore, which is very possible if the protein binds proximal to the fluorophore^65,66^. Given the small size of the oligos used in the KRIPP11 binding assay, the increase in G_12_-TAMRA-3′ fluorescence emission intensity is not surprising. However, since the 5′-FLUO-G_12_ is the source of fluorescence excitation for the G_12_-TAMRA-3′ in the FRET experiment, the G_12_-TAMRA-3′ emission would reduce or disappear in the event of quadruplex disruption regardless of the altered microenvironment. Thus, it appears that KRIPP11 does not in fact disrupt G-quadruplex structures upon binding. Taken together with the binding assays of RNAs with varying thermal history and therefore presumed quadruplex content (see above), this data suggests the possibility that G-tracts bind to KRIPP11 in quadruplex form. Given that all but one pan-edited transcript contain multiple long G-tracts (Table S3), these structures could plausibly arise in intramolecular fashion *in vivo*, in contrast to the intermolecular structures presumed to form in the *in vitro* experiments herein.

## Discussion

The family of pentatricopeptide proteins is significantly expanded in the evolutionary lineage leading to trypanosomatid parasites. Since these parasites are also characterized by numerous unusual features in RNA metabolism, including prolific U insertion/deletion editing in mitochondrial transcripts, it is tempting to speculate that the expansion of the PPR gene family in trypanosomatids represents a molecular-level adaptive radiation, in which ancestral PPR protein(s) have evolved a variety of RNA binding specificities and/or accessory domains to play distinct roles within the organelle. As one of the smallest and simplest PPR proteins in *T*. *brucei*, and one without any obvious accessory domains, KRIPP11 was our initial target for detailed characterization of RNA-binding specificity as a window into biological function. In this work, we found that KRIPP11 has a strong preferential affinity for non-duplex poly(G) sequences. KRIPP11 binds sequences of four to six or more Gs with high affinity, displays strong discrimination against other homooligomers and related sequences as well as double-stranded RNA, and shows modest specificity for RNA over identical sequences of single-stranded DNA. Bioinformatic analyses of *T*. *brucei* mitochondrial RNAs indicate a dramatic selective enrichment of G-tracts with at least four consecutive guanosines only in pre-edited transcripts of extensively edited mRNA genes (Fig. 6: Supplementary Tables S3–S7).

The simplest explanation of the above results would be that KRIPP11 interacts with transcripts destined for extensive editing and protects, sequesters, and/or targets the transcripts to the editing apparatus. Such functions are familiar roles for PPR proteins in other organisms^21–24,34–36,67,68^. Proteomic analyses of trypanosome mitochondrial ribosomes, however, indicate that KRIPP11 is associated with the small ribosomal subunit^14,31^. Indeed, a total of 20 PPR proteins have been identified as components of either the large or small subunit of the mitochondrial ribosome. Although many of these PPRs are presumed to play a structural role, some have been shown to be involved in translational activation of the mitochondrial mRNAs.

The low affinity of KRIPP11 for U-rich and A-rich RNA suggests that direct recognition of rRNA or fully-edited, translation-competent mRNA by this protein is not likely. A variety of functions for a precursor/pre-edited RNA binding protein associated with the small ribosomal subunit^31,32^ are possible. KRIPP11 binding could bridge mRNA maturation and translation, either by upregulating the substrates for the ribosome by stabilizing polycistronic precursors and pre-edited transcripts or by directly bringing the small ribosomal subunit into contact with mRNA. Alternatively, KRIPP11’s differential affinity for G-tracts of different sizes could specify the residence time of some RNA processing enzymes on their sites of action, which in the case of RNA processing nucleases could prevent stray RNA damage. By masking potential sites of spurious translation initiation, KRIPP11 could prevent premature assembly of the complete translational machinery on unedited mRNA. Evidence from plants^34–36,39,68–71^, humans^72–74^, yeast^75^, and even trypanosomes^26–28^, among others, shows that PPR proteins are capable of executing any of these functions. A more speculative function for KRIPP11 may be suggested by the existence of an alternatively-edited product of cytochrome oxidase subunit III (COIII). In this transcript, a proposed alternative editing event brings an open reading frame (ORF) found in the pre-edited 5′ end into frame with the ORF generated by editing of the 3′ end of COIII. The resulting alternatively-edited mRNA codes for the AEP-1 protein, which is essential for mitochondrial DNA maintenance^76,77^. A substantial portion of the translation-competent AEP-1 mRNA therefore contains G-tracts that are potential binding sites for KRIPP11. Similar alternative editing and dual coding events are widespread in kinetoplastid mitochondria^78,79^, and KRIPP11 may participate in stabilization or translational activation of such nonstandard mRNAs via their retained G-tracts. Further insight into the precise mechanistic role of KRIPP11 in organellar RNA metabolism will be obtained from knockdown and *in vivo* RNA-protein crosslinking studies, now in progress.

In the RNA binding assays, KRIPP11 preferentially interacted with G-tracts prepared under conditions favoring the quadruplex conformation over those in random coil states, raising the possibility that quadruplex forms may be the native target of the protein in its biological context. In recent years, it has become increasingly clear that G-quadruplex structures play important functional roles in both DNA and RNA, with specific protein targeting of RNA quadruplexes with concomitant stabilization or destabilization of the quadruplex often a key factor^80–82^. Quadruplex-containing structures have also been found in selected aptamers targeting biologically relevant proteins^83^. Thus, G-quadruplex recognition has the ability to modulate biological function in a variety of contexts. G-tracts in *T*. *brucei* pre-edited mRNAs have been shown to form G-quadruplex structures^84^, consistent with the analysis of KRIPP11 specificity shown here. If KRIPP11 does indeed bind G-tracts in their quadruplex form, one interesting unanswered question is how the structural mode of binding of PPR proteins, which in other contexts recognize non-quadruplex sequences in extended conformation^45–47^, is adapted to the very different overall shape of quadruplex RNA.

## Methods

### Materials

RNA oligos labeled with FLUO (fluorescein) at the 5′ end or TAMRA (tetramethylrhodamine) at the 3′ end were synthesized by Thermo Scientific’s Dharmacon RNA Technologies (Lafayette, CO) and deprotected according to the manufacturer’s instructions. Dry RNA was dissolved in MQ-H_2_O. 5′-FLUO labeled dG_12_ ssDNA was obtained from Integrated DNA Technologies (CoralVille, IA). The pMalTEV-E30 plasmid vector was a kind gift of Dr. Alice Barkan (University of Oregon).

### Overexpression constructs and mutagenesis

To achieve soluble expression, we fused KRIPP11 to the C-terminus of the maltose binding protein (MBP) in a pMalTEV-E30 plasmid vector^85^ using the BamH1 and SalI restriction sites (Table S8). The pMalTEV-E30 vector encodes a TEV protease cleavage site just before the BamH1 restriction site. Initial expression of MBP-KRIPP11 resulted in significant leaky expression of MBP, which contaminated preparations purified on amylose alone. Thus, we inserted a His_6_ tag at the C-terminus of KRIPP11 (Fig. 1B) to aid tandem metal ion- and amylose affinity chromatography. MBP-His_6_ (used as a negative control) was expressed from a construct created by inserting His_6_ at the C-terminus of MBP just before the TEV protease cleavage site (Fig. 1C) followed by conversion of the first residue of KRIPP11 in MBP-His_6_-KRIPP11 to a stop codon. Mutagenesis was performed by PCR following the QuikChange^**®**^ procedure using the primers indicated in Table S8.

### Protein expression and purification

Protein was expressed in BL21(DE3) *E*. *coli*. Recombinant colonies were inoculated into 10 ml LB broth and grown overnight at 37 °C and 250 rpm. 20 ml of this seed culture was then added to 980 ml of LB broth, grown at 37 °C, 250 rpm to an OD_600_ of about 0.7, induced with 0.1 mM isopropyl-β,D-thiogalactopyranoside (IPTG), and maintained at 22 °C for 6 hours. Cultures were harvested by centrifugation at 4 °C followed by protein purification under native conditions. Bacteria were resuspended in lysis buffer (50 mM NaH_2_PO_4_, pH 8.0, 300 mM NaCl, 10 mM imidazole, 5 mM β-ME), lysed by a microtip sonicator and clarified by centrifugation. The supernatant was loaded onto a Ni-NTA column and MB-KRIPP11-His_6_ was eluted by 50 mM NaH_2_PO_4_, pH 8.0, 300 mM NaCl, 200 mM imidazole, 5 mM β-ME. Ni-NTA eluates were immediately repurified using an amylose column. Amylose eluates were then dialyzed against at least 40 volumes of 10 mM Tris-HCl, pH 7.5, 50 mM NaCl at 4 °C for at least 12 hours with a 7 K MWCO SnakeSkin® tubing (Thermo Scientific, Rockford, IL). Dithiothreitol (DTT) was then added to the dialysates to 1 mM followed by concentration using 10 K MWCO centrifugal filters. Protein purity was assessed by 5–12% SDS-PAGE and nucleic acid contamination was monitored by the A_260_:A_280_ ratio.

### KRIPP11 native gel electrophoresis

After detachment of MBP by TEV protease, free KRIPP11 aggregated upon purification even in the presence of detergents and glycerol. To establish the oligomeric state of KRIPP11, we therefore performed blue native PAGE on the TEV protease cleavage mixture of MBP-KRIPP11-His_6_. Briefly, MBP-KRIPP11 was purified by tandem Ni-NTA and amylose as above. Dilute protein was then concentrated to 0.7 mg/ml followed by TEV protease cleavage in the presence of 50 mM Tris-HCl, 0.5 mM EDTA, 1.0 mM DTT, plus or minus 0.05% (w/v) DDM. The reaction was performed at 8 °C for 13 hours. 1.6 µl of 10x blue native Tris-glycine (BNTG) sample buffer (250 mM Tris, pH 7.0, 30% (w/v) sucrose, 5% (w/v) brilliant blue-G) was then added to 14.4 µl of TEV protease cleavage mixture and run on a 6–12% polyacrylamide gradient resolving gel with a 3.5% polyacrylamide stacking gel at 150 V constant voltage, 4 °C, for one hour. 25 mM Tris, 250 mM glycine was used as running buffer. Native TEV protease (27 kDa), thioredoxin-His_6_ (17 kDa), MBP (45 kDa), MBP-KRIPP11 (69 kDa), and factor Xa protease (43 kDa) were the size markers.

### Electrophoretic mobility shift assays (EMSA)

5′-FLUO labeled RNA and DNA oligonucleotides were used, namely, single stranded C_12_, U_12_, A_12_, G_12_, G_9_, G_6_, G_6_C_2_U_2_A_2_, G_4_, G_4_C_2_U_3_A_3_ and (GGU)_4_), and double stranded (G.C)_12_. Double-stranded (G.C)_12_ was prepared by heating a mixture of G_12_ ssRNA, a three-fold excess of C_12_ ssRNA, and a 5x annealing buffer (50 mM Tris-HCl, pH 7.5, 250 mM NaCl, 5 mM EDTA) on boiling water for two minutes followed by slow cooling at room temperature for one hour. Agarose rather than polyacrylamide gels were used for electrophoresis because the latter were associated with strong well retention of the RNA as the protein concentration rose. Due to the thickness of the agarose gels and the propensity of poly(G) to quench fluorescein, 150 nM RNA/DNA was required in the reaction for optimal signal in the gel images. The RNA binding reaction consisted of 2.5 µl of RNA/DNA, 7.5 µl of protein (MBP-KRIPP11-His_6_ or MBP-His_6_ control as labeled), and 2.5 µl of a 5x RNA binding buffer adapted from Stuart and coworkers (100 mM Tris-HCl, pH 7.5, 750 mM KCl, 25 mM MgCl_2_, 5 mM DTT, 0.5 µg/µl BSA, 2 U/µl RNAsin)^86^. 25 mM Tris, 250 mM glycine was used as running buffer. Gels were scanned using a VersaDoc™ MP 4000 fluorescence imager (Bio-Rad Laboratories).

### Fluorescence polarization spectroscopy

The anisotropy of 20 nM 5′-FLUO labeled ssRNA or ssDNA was assessed in the presence of increasing KRIPP11 concentration. A 450 µl reaction for each protein concentration was prepared from dialysis buffer, MBP-KRIPP11-His_6_, 45 µl of a 10x binding buffer (200 mM Tris-HCl, pH 7.5, 1.5 M KCl, 50 mM MgCl_2_, 10 mM DTT), and 50 µl of 180 nM RNA/DNA. After at least 15 minutes of equilibration at room temperature, the intensities of vertically- and horizontally polarized fluorescence emission (I_vv_ and I_vh_ respectively) following vertically polarized excitation were obtained at 25 °C using a 5 mm microsquare quartz sample cell in a QuantaMaster spectrofluorometer (Photon Technology International). Noise from Raman scattering was removed by subtracting the fluorescence emission of KRIPP11 dialysis buffer from data. For all assays, the instrument grating factor (G) was about 1.5. Excitation was at 450 nm with a 2.0 mm slit with emission measured at 520 nm with a 3.8 mm slit. Fluorescence anisotropy was computed from:

1 $$r=\frac{{I}_{vv}-G{I}_{vh}}{{I}_{vv}+2G{I}_{vh}}$$

The mean anisotropies of at least three data sets were then normalized and used to determine the K_d_ by nonlinear fitting with IGOR Pro 6 according to

2 $$r=\frac{nc}{{K}_{d}+c}$$

where c is the protein concentration and *n* is an overall scaling factor.

Preliminary experiments indicated stronger and more reproducible interactions of KRIPP11 with slowly thawed G-tract RNA than with either rapidly thawed or thermally annealed RNA (cf. also Fig. 7A). Thus, reported anisotropy experiments used RNA that was thawed slowly on ice-cold water.

### *In vitro* selection of consensus RNA ligands for KRIPP11

RNA aptamers for KRIPP11 were isolated from a pool of 2.4 × 10^18^ random sequences (20 nucleotide variable region) by 11 rounds of *in vitro* selection using immobilized MBP-KRIPP11-His_6_ on Ni-NTA magnetic beads using procedures based on published methods^87^. RNA was synthesized by T7 RNA polymerase from a DNA template containing a variable region of 20 nucleotides surrounded by primer binding sites followed by treatment with DNase and purification. Negative selection to remove sequences with affinity to the fusion partner was performed in every second round of selection using MBP-His_6_ as a decoy. At each round, the RNA pool in binding buffer (50 mM KH_2_PO_4_, pH 7.5, 100 mM KCl, 1.5 mM MgCl_2_, 2.0 mM imidazole) was incubated with MBP-His_6_ beads (if performed) at 4 °C for 20 minutes. The flow-through from the latter was then incubated with MBP-KRIPP11-His_6_ for 20 minutes. Binding stringency was increased across the course of the selection by decreasing protein and (for the final round) RNA concentration (Table S10). After drainage of unbound RNA, the beads were washed with binding buffer followed by recovery of bound RNA by phenol:chloroform extraction and ethanol precipitation and reconstituted in MQ-H_2_O. The resulting RNA pool was converted to cDNA with M-MLV reverse transcriptase followed by PCR-amplification, transcription, and RNA purification in preparation for the next round of selection as described previously^87^. Enrichment of KRIPP11-bound RNA after each round of selection was quantitated with the Ribogreen assay^88^ using a FLUOstar Omega microplate reader (BMG LABTECH). The cDNA from the 11^th^ round RNA was cloned into a TOPO^®^ TA plasmid and sequenced by dideoxynucleotide chain termination. Sequences were then aligned with MEME^89^ and WebLogo^90^ to identify conserved elements.

### Analysis of G-tract content in *T*. *brucei* mitochondrial transcripts

Sequences of all the kinetoplast pre-mRNA and rRNA genes were decoded from the *T*. *brucei* maxicircle DNA sequence (GenBank accession # M94286.1). Edited mRNA sequences were obtained from Hong and Simpson^91^. A census of nonoverlapping dodecanucleotides containing at least one homopolymeric stretch of four or more Gs in each RNA was then made. Next, a count of each G_n_ homopolymer (n = 4, 5, 6, 7, 8, or 11; no sequences of G_9_ or G_10_ were present) in each transcript category was made.

### Circular dichroism (CD) spectroscopy of guanosine tract RNA

The native conformation of G-tract RNA ligands was investigated by CD spectroscopy. CD spectra were collected using a Chirascan CD spectrometer (Applied Photophysics, Leatherhead, UK) and a 0.1 mm path length cuvette. To assure native conformation, frozen RNA samples in MQ-H_2_O were thawed slowly on ice-cold water. For G_12_, the concentrations of RNA used were 4.1 µM in MQ-H_2_O and 3.7 µM in RNA binding buffer (20 mM Tris-HCl, pH 7.5, 150 mM KCl, 5.0 mM MgCl_2_, 1.0 mM DTT). For G_4_, the concentrations of RNA used were 7.5 µM in MQ-H_2_O and 6.8 µM in RNA binding buffer. An average of three spectra was obtained per sample. Before CD spectroscopy, the G_12_ RNA was desalted by dialysis against two1000-fold volumes of MQ-H_2_O at 12 hour intervals for 24 hours at 4 °C using a 3.5 K Slide-A-Lyzer® cassette (Thermo Scientific). G_4_ was used in undialyzed form due to lack of retention by the molecular-weight filters used.

### RNA native gel electrophoresis

To further understand the basis for better KRIPP11 binding to native G-tracts than thermally exposed ones, we investigated the gel mobility of four samples of 5′-FLUO-G_12_ RNA with varying thermal history. One RNA sample was not exposed to elevated temperature, and represented the native RNA. The second RNA sample was heated at 95 °C for 10 minutes, then one of several different 10X RNA binding buffers (see caption to Fig. 7) was added after which tubes were transferred to 95 °C hot water and left to slowly cool to room temperature for 4 hours followed by storage at 4 °C for at least five days. The third RNA set was heated at 95 °C for 10 minutes, snap-cooled on ice for 2 minutes, transferred to room temperature, and RNA binding buffer added. The fourth RNA set was heated at 95 °C for 5 minutes, snap-cooled on ice for 2 minutes, transferred to room temperature, and RNA binding buffer added. For analysis, 12 µl of a mixture comprising 10.8 µl of 50 nM RNA and 1.2 µl of 10X sample buffer (10 mM Tris, 1.0 mM EDTA, pH 7.5, 50% (v/v) glycerol, 0.01% (w/v) bromophenol blue, 0.01%(w/v) xylene cyanol) were loaded on to a native 18% polyacrylamide (29:1 acrylamide: N,N’-methylene-bis-acrylamide) gel and run at 150 V at 4 °C using TBE (50 mM Tris, 50 mM boric acid, 1.0 mM EDTA). A 12 nt poly(A) ssRNA oligo (A_12_) was loaded as marker for monomeric G_12_ RNA.

### Fluorescence resonance energy transfer (FRET)

FRET was used to determine whether KRIPP11 binding perturbs the native conformation of G-tract RNA. Separately labeled 5′-FLUO-G_12_ and 3′-TAMRA labeled G_12_ (G_12_-TAM-3′) ssRNAs were desalted by dialysis against 200-fold volume of MQ-H_2_O at 4 °C overnight using a 2.0 K MWCO Spectra/Por® dialysis membrane (Spectrum Laboratories Inc, Rancho Dominguez, CA). To anneal the 5′-FLUO-G_12_ and G_12_-TAM-3′, a mixture of 350 µl of each RNA at 3.0 µM monomeric concentration was heated at 95 °C for 10 minutes, then 245 µl of MQ-H_2_O and 105 µl of 10X RNA binding buffer were added, diluting the RNA to 2.0 µM monomeric concentration. Tubes containing the RNA mixture were then transferred to 95 °C water in a plastic dish and allowed to slowly cool to room temperature on the bench for 4 hours. Finally, the RNA was stored at 4 °C for at least five days to allow further renaturation before FRET assays. The intensities of fluorescence emission of 50 nM monomeric concentrations of 5′-FLUO-G_12_ alone, G_12_-TAM-3′ alone, and of the annealed FLUO-G_12_/G_12_-TAM mixture in the absence and presence of various KRIPP11 concentrations were then collected at 25 °C following excitation at 490 nm as described above. Three datasets were used to compute average spectra.

## Electronic supplementary material

Supplementary Information

## Data Availability

The datasets generated and analyzed during the current study are available from the corresponding author on reasonable request.
