# Supplementary material for: The 27 kDa Trypanosoma brucei Pentatricopeptide Repeat Protein is a G-tract Specific RNA Binding Protein
Source: Sci Rep. 2018 Nov 19;8:16989. doi: 10.1038/s41598-018-34377-9 (PMC6242908; doi:10.1038/s41598-018-34377-9)
Supplement: Supplementary file 1 — Supplementary Information [file 41598_2018_34377_MOESM1_ESM.pdf]

SUPPLEMENTARY INFORMATION

for

The 27 kDa *Trypanosoma brucei* Pentatricopeptide Repeat Protein is a G-tract Specific RNA Binding Protein

Pakoyo F. Kamba[1,2,4], David A. Dickson[1], Neil A. White[1], Jennifer L. Ekstrom[1], Donna J. Koslowsky[2], Charles G. Hoogstraten[1*]

[1]Department of Biochemistry and Molecular Biology; [2]Graduate Program in Cell and Molecular Biology; [3]Department of Microbiology and Molecular Genetics, Michigan State University, East Lansing, Michigan, 48824-1319, USA; [4]Makerere University, Kampala, Uganda.

Table S1. Prediction of organellar localization of KRIPP11 by a variety of contemporary algorithms.

Localization tool	Targeting prediction
pTARGET	Mitochondrial
TargetLoc	Mitochondrial
DBSubLoc	Mitochondrial
SLPFA	Mitochondrial
Phobius	non-cytoplasmic
ESLPred	Mitochondrial
LOCtree	mitochondrial
CELLO: by amino acid composition	Mitochondrial
CELLO: by N-terminal peptide	non-mitochondrial
SHERLOC: by amino acid composition	Mitochondrial
SHERLOC: by N-terminal peptide	non-mitochondrial
pLOC: by amino acid composition	Mitochondrial
MultiLoc: by amino acid composition	non-mitochondrial
MultiLoc: by N-terminal peptide	Mitochondrial
TargetP 1.1	None
MitoProt II	None

Table S2. Randomized regions of sequenced clones from *in vitro* selection experiments targeting KRIPP11. The randomized regions are flanked by 5´-AA and 3´-GA dinucleotides.

Random region sequence[a]	Identified homooligomers
GGUAUAUAUGUUAGCUGGUC	
UCGGGCGGCUGAGGGUUCCC	
UCAGUACGUCUCCAUUGUUU	
AGGUAGCGGUGGUGGGAGCC	
CUCUAUGGGGCGUGUCAUUGA	G4
AGGCGGACAACUCGUGUUUG	
GGAUUUUCUUGGACCUCCAC	U4
GCUCGACCAAUUUUAGUUGU	U4
GAAAACGGGUGAGGUAAAUA	A4
UUGUAUGGUACACCGUUGGU	
UCGACUGAUUAAGAAGCGUU	
CGUCGGUACUUGCGCGUGCA	
GUGGCUCCAUACGAUUAAGG	
GGUGCUCCACGUCUCUUUG	
CGAGCUUGGCUUGGUCCUAC	
CAUCUAACGACAAUCGUCUG	
AUUUAGUGUCAACACGGUCA	
UAGUCGUUGCUGGAGGUUUU	U4
CAUCGUUUAAGAUCGGCUAC	
UGUUACCUGCUGUGUCAACC	
CGGUCGGUAGUUUAGGGAGA	
GGUUUGAAUUAAGUUUAUC	
GGCCACGCACUGAGACUCGGU	
GUUGUCGUGGUUCCGUACGG	
AUCGGGGCCAGGGUUGGUUG	G4
UUCUCGUGUCUCCGGGUGGU	
CGGGGUGGCAUUCGGGGGGG	G4; G8[b]
AAGUGGUAGGUAGUCUGGAC	
AAGUGGUAGGUAGUCUGGAC	

UGGGUGCUGUUGGCCCGUCU	
GUGGCCUAGCACCCGCGGGU	
UCACUCUGAUCGAAUUCGCU	
UGUAUCCCGAAAAGAAUAU	A4
AAUGGAUGUUUUGCAUAAU	U5
CGGUGCUUCUGUCGAGCCUUU	
GUCAAGAGACGUGUUAUUGA	
CACUAUGUCCAGCACUGUUAU	
CUUUGACUUCAGCCUCACUU	
CGGUUGUGCGUUAUUGGUCC	
GAUCUGAUUGCGUGCGUGCU	
GGAGUGCUAGUUUCCCCUCG	C4
UUGGAAUAUAUUCCUCCAGC	
GUCCGUCCUUCGUCUGGUGU	
UGUUAGCUUGUUUAUCCCUC	
GACUGCCGUUAAAGGAUGGU	
GGCCUGUACUCCUCUUUUGA	U4
CUAACGAUUUUCUACAUGGG	U4; G4[b]
ACUCUAGUGGAAUUCGUAUG	
CGUCUUUGCGAGCAGCCACG	
UAAACCCUGGCUGGUUGCGU	
UCGUGGGGGAAGACUUGAAU	G5
GACUGCCGUUAAAGGAUGGU	
GGCCUGUACUCCUCUUUUGA	U4
CUAACGAUUUUCUACAUGGG	U4; G4[b]
CAUCUUAGAGCCGGUGUUCC	
CGAUGGCCGUACGAUAGACG	
CGAUGGCCGUACGAUAGACG	
CAAGAGGUGUCGGGGUAAAG	G4
UGUCUUAUUGUAUUUACAUG	
UAGCUCAUGCCUGGUUAUUU	
GUUCUUCGAAUGGGGCUAGA	G4

GAACGAGUCCCACUCGGCCG	
CUACGUUUUCAUACUCUCGG	U4
CUGCGGGUUGAAUGCAUUAU	
UUAUGCCCUUGCGAUCGCUC	
GAUGCCCCCUGUCGGAUGUU	C5
UUCAAUUGUUUGUGGAGACA	
CUAAGAACUGGAUUUGAUAG	
ACUGGAUGCUUUUCGGCUAG	U4
CUAAGAACUGGAUUUGAUAG	
GGUUUUGGGUUGUUCGUAAU	U4
UUGUUAAUGCCUUAGGGAUG	
GGCUUCUUGAACGCAAUACU	
ACUGUAAUCGUAGGUGACUG	
UUACUGCUUCGAAAUGAGAC	
GGCAUGUAGUUGAAACUGAG	
CUGGUGAAAGGAGGGGUGAU	G4
CUGAGAUAAGCUAGAUCAC	
CUUCUAACAGUGGGUUGGUC	
CAAAGUGGCGUUAGUAAGGG	
UUUCACAUUAAGUCCUGCUG	
UGGGUCAUCUAACUGGCUAG	
GCUGUACAGUAUACUAGAUU	
AUGCUUAUGUAGAUCUACUU	
CAUAGAGUACUAGUAAUUGA	
GCUGCUGAGAAUCUGCUCUC	
GACGAGACCGGCUCGUCUGG	
AGGGCCCGUAUCUUGUUAAA	
AUAAGACGGCUGUGUGAAUG	
CUAUUGCAGGGUGUUGAGA	
UCUGAUAUGUCUCUGUGUUA	
GAAGGCGUAGCCUAAUCCUG	
GGAUGGUAUUGAGUCAUCAU	

UAAGAUAAACGCAUGAUUGC	
CGUUGUAAGGGGUUCAAUGU	
CGUUGUAAGGGGUUCAAUGU	
CGGCGCUGAUCGUAUGAGAG	
UUGAGGGAGCGGAUAUAGGU	
CGUUGUAAGGGGUUCAAUGU	
AUCCCCUUGUAUUGGCCCGU	C4

[a] Random regions of 19 and 21 nucleotides represent single-base deletions and insertions during the selection process, respectively.

[b]Includes 3´-G from the constant region.

Table S3. Non-overlapping 12-mer guanosine RNA tracts (G-tracts) with at least four consecutive guanosines in *T. brucei* pre-edited transcripts of pan-edited mRNA genes.

Gene	G-tract	Number of Gs within tract	Longest poly(G)
1) ND7	GGAGGAGAGGGG	9	4
	GAGGGGAAGAGC	7	4
	CCGAGAAGGGGG	7	5
	GAGGGGAAGGGG	9	4
	GGGGCGAGCAGG	8	4
	GAGGGGGAGGGG	10	5
	GAGAGAGAGGGG	8	4
	GCGGCGGGGCAG	8	4
	GGGGGCCGCGAG	8	5
	GAGGGGAGAGUC	7	4
	GGGGGGGGGGGA	11	11
	GGGGGGGGCCGG	10	8
	GAGGAAUGGGGG	8	5
	GAGGGGACCGUA	6	4
2) ND8	GGAAGGUGGGGA	8	4
	GGGGGAGAGCGG	9	5
	GGGGGGGAGGGG	11	7
	GGAAGGGGAGCA	7	4
	GGAGGGGAGCCA	7	4
	GAGGGGGAGAGA	8	5

	GGGGAGAAGGGG	9	4
3) ND9	GGGGAGAGGGUU	8	4
	GGGGAGAGGAGG	9	4
	AGGGGGCGAGGG	9	5
	GGGGCGGGGGGG	11	7
	GCGGGGGAACGC	7	5
	GAGGAGGGGGGG	10	7
	GGAUCCAAGGGG	6	4
	GGGGGGGAGGAG	10	7
4) COIII	AAGGGGAGGGGG	9	5
	GGAGGAGGGGGA	9	5
	AGGGGAGGGGAG	9	4
	GGAGAGGGGAGG	9	4
	GGAGGGGUUGGG	9	4
	GAGAGGGGGGGG	10	8
	GGGGUGGGCAAA	7	4
	GAGGGGGGAGAG	9	6
	CGGGGGAAAGGG	8	5
5) ATPase A6	GGGGGGGAGGGG	11	7
	GGGGAAGAGGAG	8	4
	GGGGAGAGGCGG	9	4
	GGAUAAGAGGGG	7	4
	AAGGGGAAAUGG	6	4

	GGGGGAGGAGAG	9	5
6) CR3	AAGGAUUGGGGG	7	5
7) CR4	GGGGCAAGGGUG	8	4
	AGGGGGGUUUGU	7	6
	GGGGGAGAGGAA	8	5
	GGGGGUUUGGGG	9	5
	GAAGGGGAGAAG	7	4
	AAAUUGAAGGGG	5	4
	UUGAUUGGGGGG	7	6
	GGGGAGAAAGUG	7	4
	GGGGUGGGGGAG	10	5
	GGGGAGAGGGGG	10	5
8) ND3	GGGGGGCGGGGU	10	6
	GGGGUGAAGGGA	8	4
	GGGGGGAGAAGG	9	6
	GGGGAGGGAUCA	7	4
9) RPS12	GGGGACGGAGAG	8	4
	GGGAGGCGGGGA	9	4
	GAGGGUGGGGGG	10	6

Table S4. Non-overlapping 12-mer guanosine RNA tracts (G-tracts) with at least four consecutive guanosines in *T. brucei* pre-edited transcripts of limited-editing mRNA genes.

Gene	G-tract	Number of Gs within tract	Longest poly(G)
1) Cyb	AUAUGGGGUAGG	6	4
	GGGGAAGUGAAU	6	4
2) COII	NONE		
3) MURF2	NONE		

Table S5. Non-overlapping 12-mer guanosine RNA tracts (G-tracts) with at least four consecutive guanosines in *T. brucei* edited transcripts of both pan-edited and limited-editing mRNA genes.

Gene	G-tract	Number of Gs in tract	Longest poly(G)
1) ND3	NONE		
2) ND7	NONE		
3) ND8	NONE		
4) ND9	NONE		
5) ATPase A6	NONE		
6) COIII	NONE		
7) CR3	NONE		
8) CR4	NONE		
9) RPS12	NONE		
10) Cyb	UAUGGGGUAGGU	6	4
	UUUGGGGAAGUG	6	4
11) COII	NONE		
12) MURF2	NONE		

Table S6. Non-overlapping 12-mer guanosine RNA tracts (G-tracts) with at least four consecutive guanosines in transcripts of *T. brucei* never-edited mitochondrial mRNA genes.

Gene	G-tract	Number of Gs in tract	Longest poly(G)
1) MURF5	NONE		
2) MURF1	NONE		
3) ND1	NONE		
4) COI	UGGUUUUUGGGG	6	4
	GUUGGUUGGGGG	8	5
5) ND4	NONE		
6) ND5	NONE		

Table S7. Non-overlapping 12-mer guanosine RNA tracts (G-tracts) with at least four consecutive guanosines in transcripts of *T. brucei* mitochondrial rRNA genes.

Gene	G-tract	Number of Gs in tract	Longest poly(G)
1) 12S rRNA	GUUUGAUUGGGG	6	4
2) 9S rRNA	NONE		

Table S8. Primers used in cloning and site-directed mutagenesis.

Activity	Primer code	Primer sequence (5' to 3')
Add BamH1 restriction site on to 5' end of KRIPP11	CGH30	ACTTCCAGGGATCCGGTCACGTGTACGCCCTTC
Add SalI restriction site on to 3' end of KRIPP11	CGH34	GCCTGCAGGTCGACTCAACCACGAGGTAAAGT
Insert His_6 tag at C-terminus of KRIPP11	CGH69_F	CCACTTTACCTCGTGGTCACCACCACCACCACCA-CTGAGTCGACCTGCAG
	CGH70_R	CTGCAGGTCGACTCAGTGGTGGTGGTGGTGGTGA-CCACGAGGTAAAGTGG
Insert His_6 tag between MBP and KRIPP11ΔNR2[a]	CGH67_F	GGATTTCAGAATTCGGATCTCACCACCACCA-CCACCACGAAAACCTGTACTTCCAGGG
	CGH68_R	CCCTGGAAGTACAGGTTTTCGTGGTGGTGGT-GGTGGTGAGATCCGAATTCTGAAATCC
Convert MBP-His_6-KRIPP11-ΔNR2[a] to MBP-His_6	CGH77_F	TACTTCCAGGGATCCTGATGCGCTCTTGCAGCC
	CGH78_R	GGCTGCAAGAGCGCATCAGGATCCCTGGAAGTA

[a]A plasmid containing a truncated form of KRIPP11 lacking the first two N-terminal PPR motifs (hence ΔNR2) was used as template for creation of MBPHis_6.

Table S9. Stringency conditions for successive rounds of *in vitro* selection.

Round	[RNA] (μM)	[MBP] (μM) Counterselection	[PPR] (μM) Selection
1	18	-	8.02
2	30	3.00	2.05
3	15	-	3.14
4	30	3.04	2.19
5	30	-	3.21
6	30	3.10	0.64
7	30	-	2.18
8	30	2.11	2.09
9	30	-	1.11
10	5	0.50	0.83

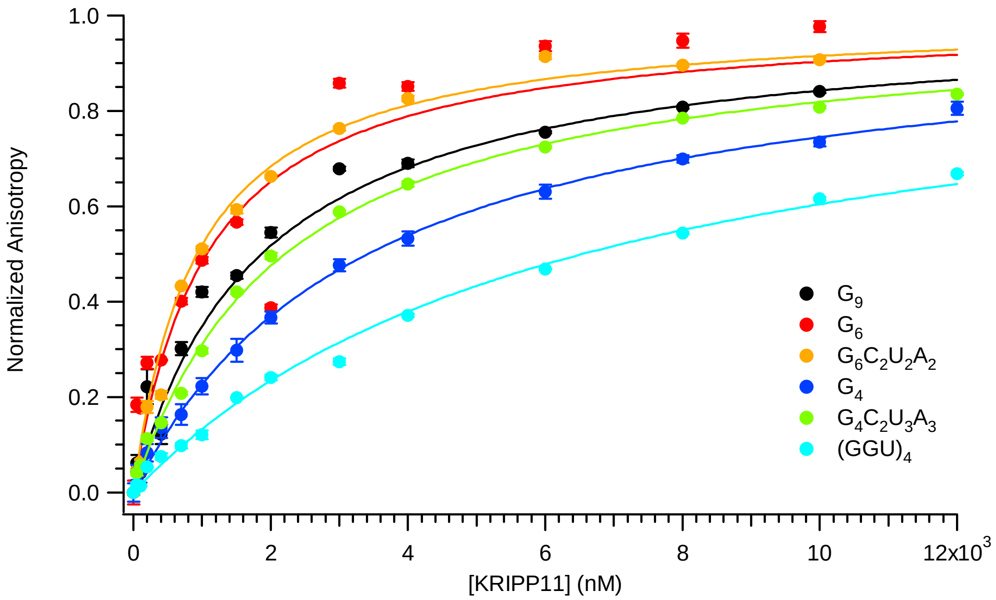

Figure S1. Anisotropy of interaction of KRIPP11 with RNA G-tracts having fewer than 12 guanosines. Each reaction contained 20 nM 5’-FLUO labeled ssRNA, RNA binding buffer (20 mM Tris-HCl, pH 7.5, 150 mM KCl, 5.0 mM $MgCl_2$, 1.0 mM DTT), and MBP-KRIPP11.

Figure S2. Original images for all gels. In the pages that follow, labels refer to the Figure and panel in the main text.

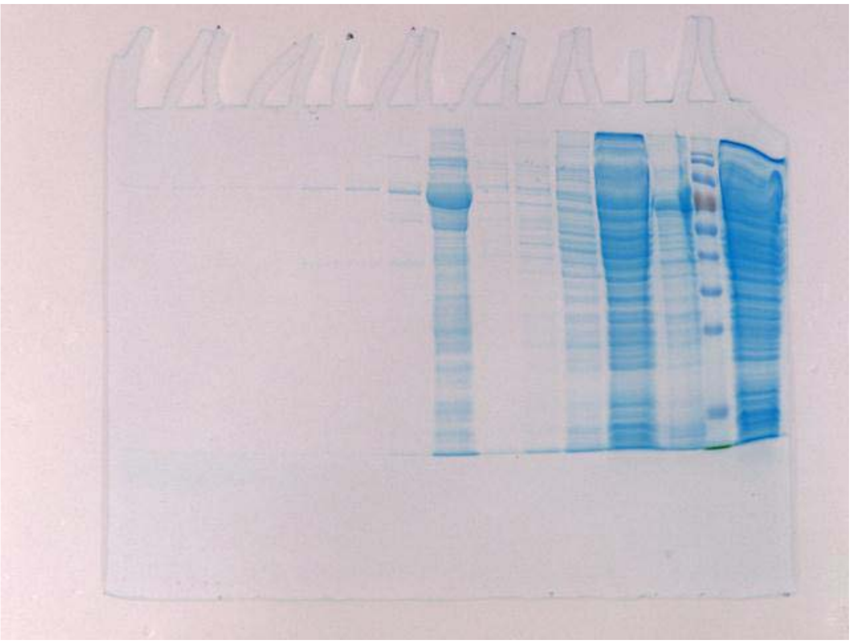

Figure 1D

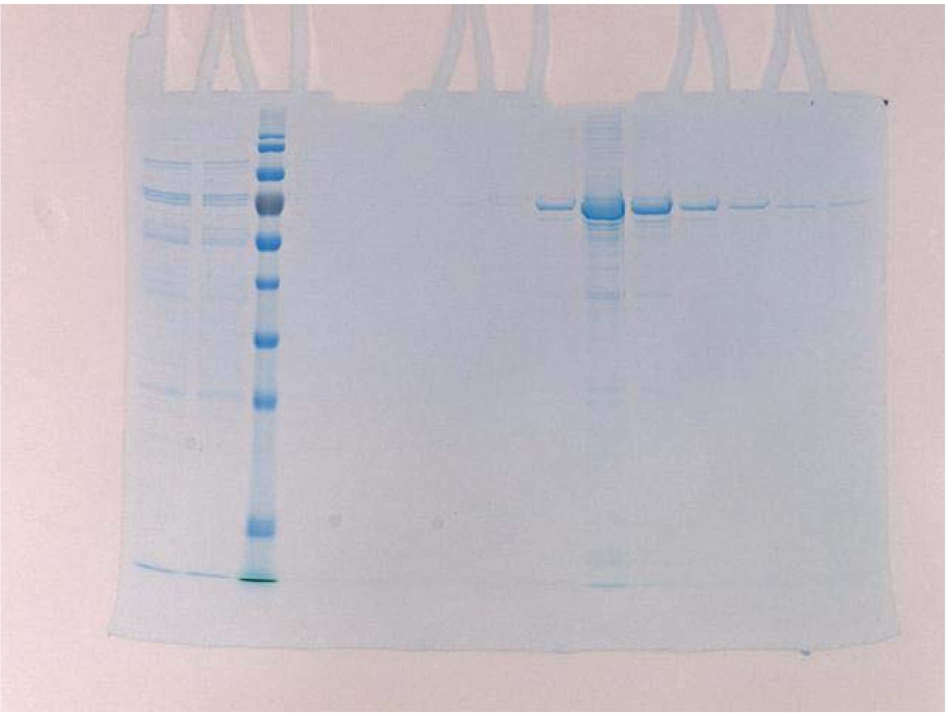

Figure 1E

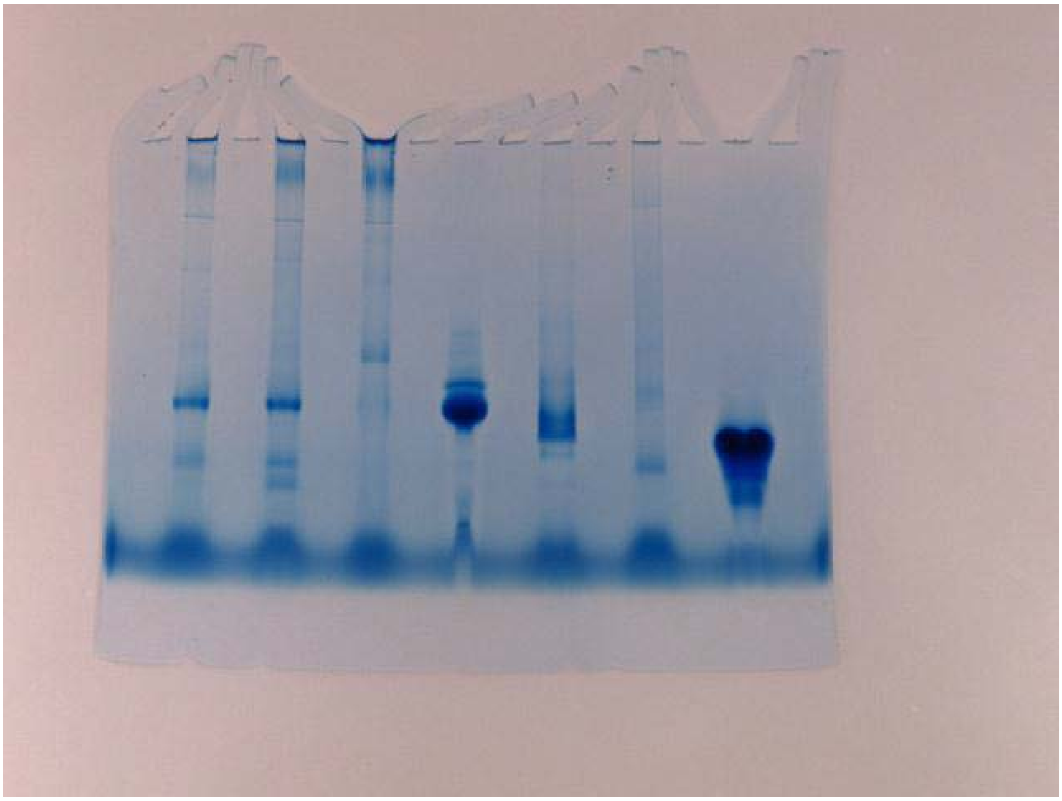

Figure 1F

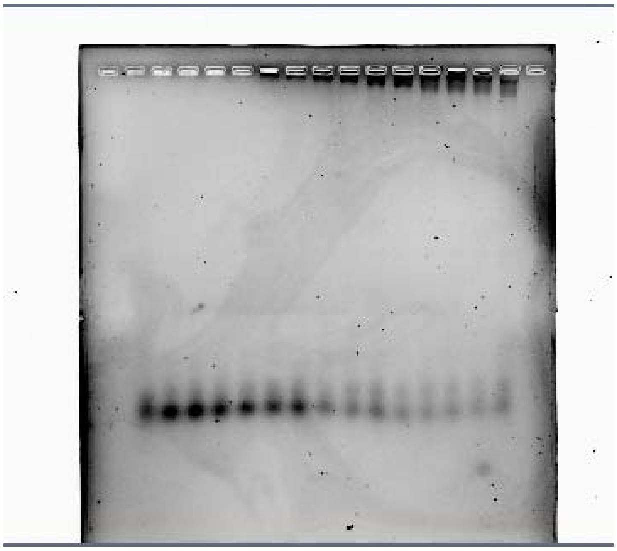

Figure 2A

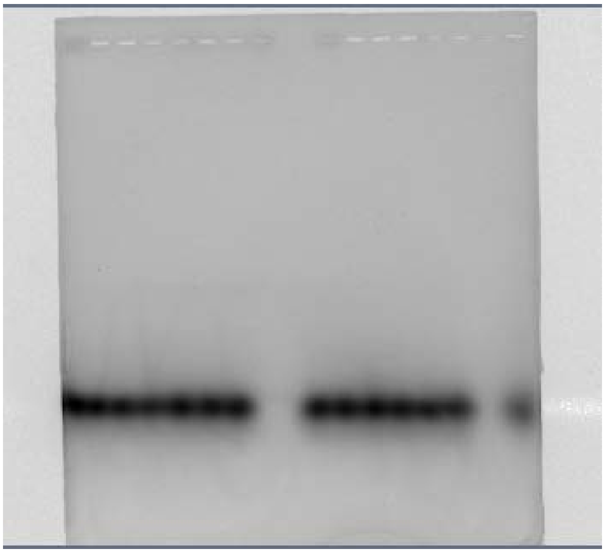

Figure 2B

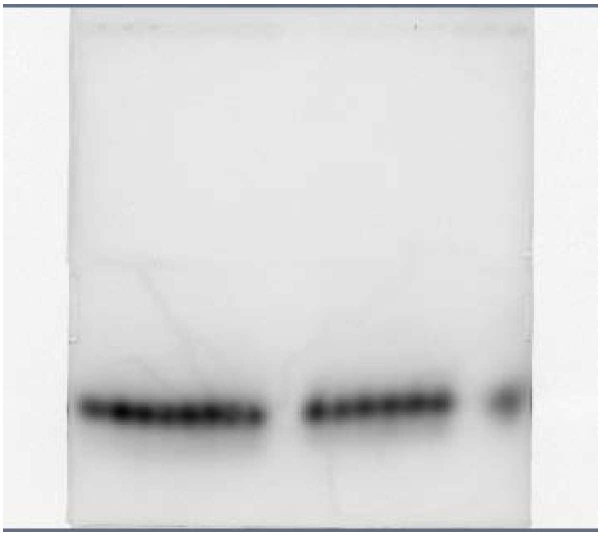

Figure 2C

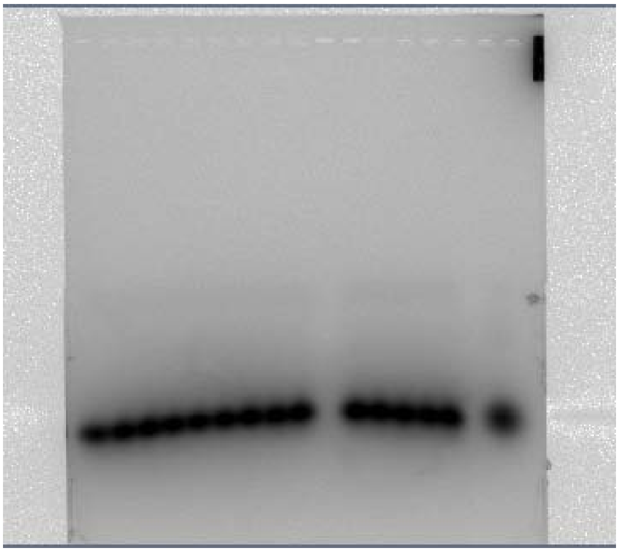

Figure 2D

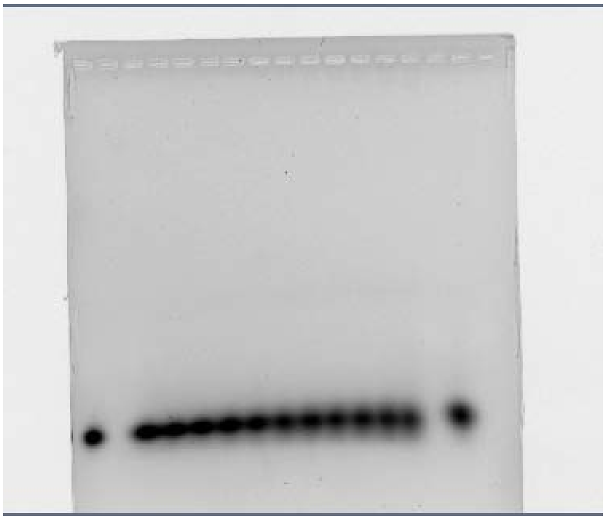

Figure 2E

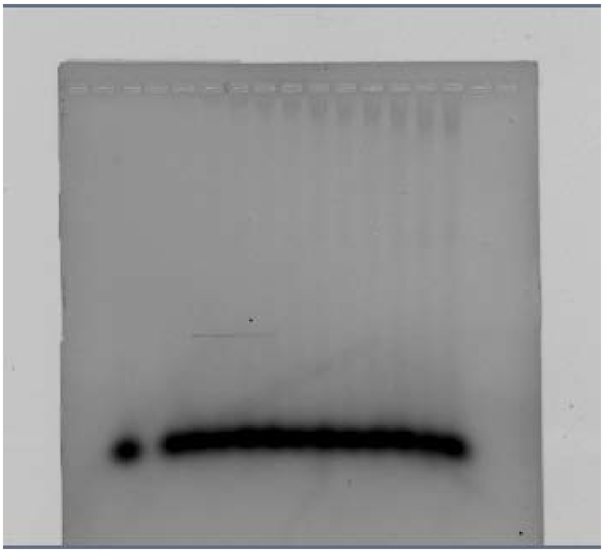

Figure 2F

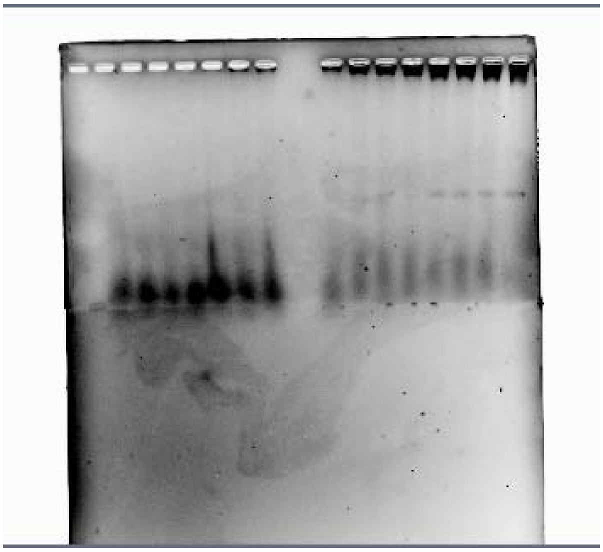

Figure 3 G_9

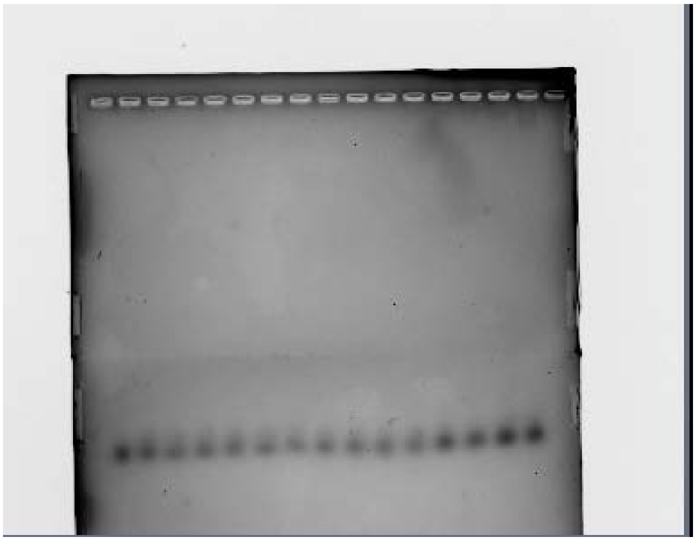

Figure 3 G_6

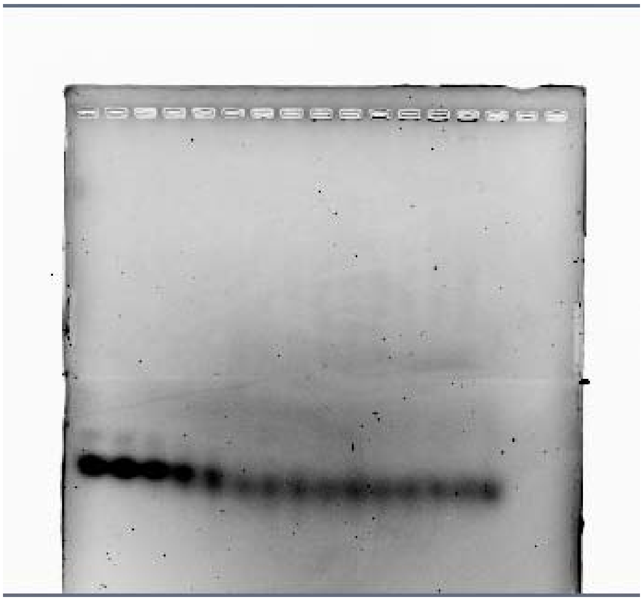

Figure 3 $G_6C_2U_2A_2$

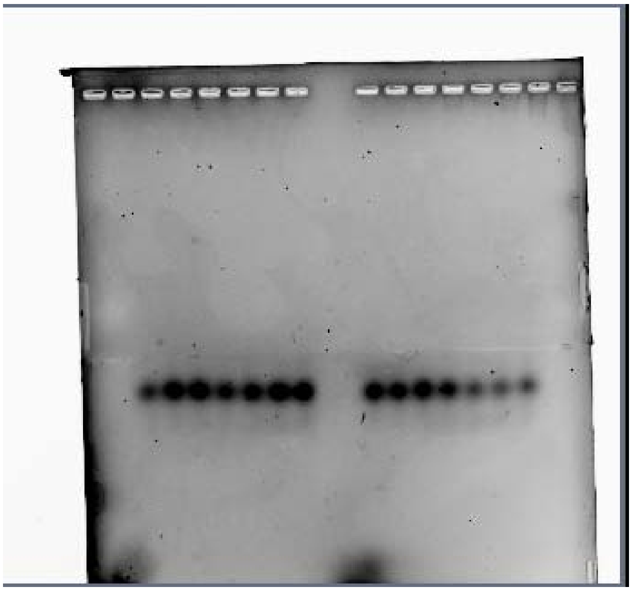

Figure 3 G_4

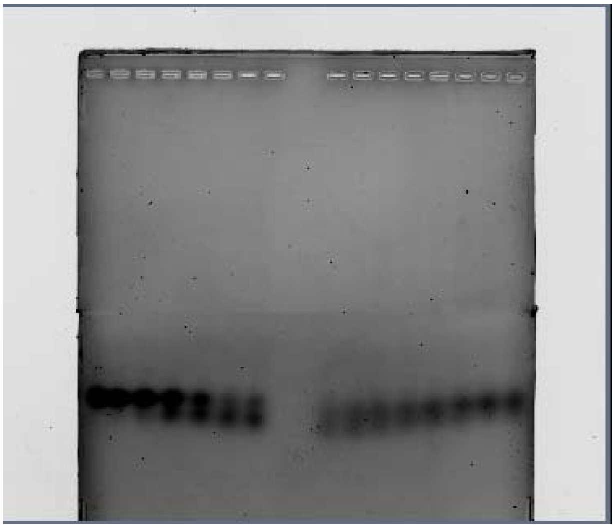

Figure 3 $G_4C_2U_3A_3$

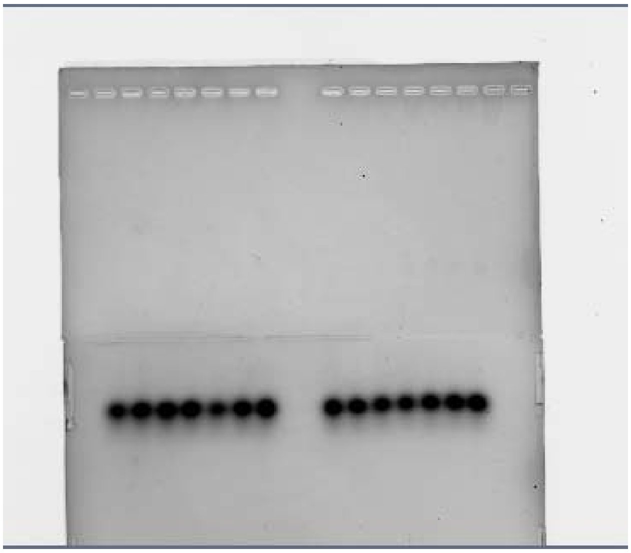

Figure 3 $(GGU)_4$

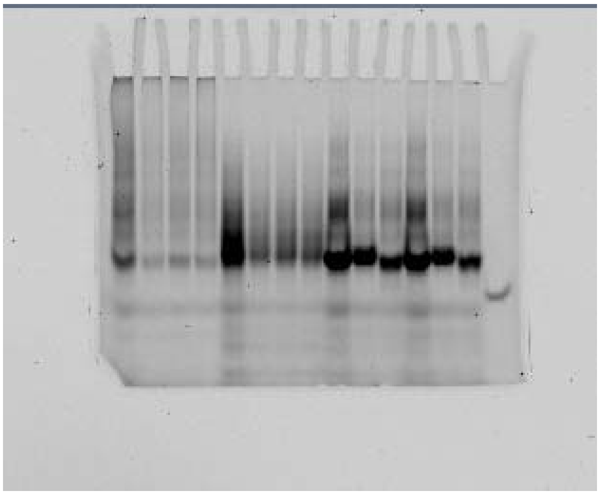

Figure 7B

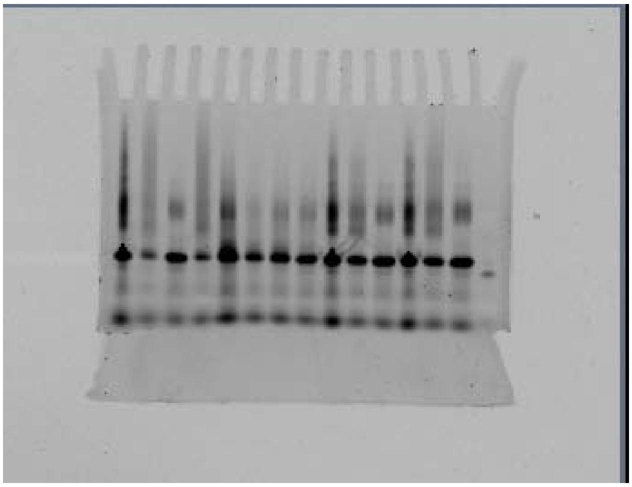

Figure 7C
